# Dentition and feeding in Placodontia: tooth replacement in *Henodus chelyops*

**DOI:** 10.1186/s12862-021-01835-4

**Published:** 2021-07-05

**Authors:** Yannick Pommery, Torsten M. Scheyer, James M. Neenan, Tobias Reich, Vincent Fernandez, Dennis F. A. E. Voeten, Adrian S. Losko, Ingmar Werneburg

**Affiliations:** 1 Senckenberg Centre for Human Evolution and Palaeoenvironment (HEP) an der Eberhard-Karls-Universität Tübingen, Sigwartstraße 10, 72076 Tübingen, Germany; 2 Fachbereich Geowissenschaften, Eberhard-Karls-Universität Tübingen, Hölderlinstraße 12, 72074 Tübingen, Germany; 3 Université de Bourgogne-Franche-Comté, Esplanade Erasme, 21000 Dijon, France; 4 Universität Zürich, Paläontologisches Institut und Museum, Karl Schmid-Strasse 4, 8006 Zürich, Switzerland; 5 Oxford University Museum of Natural History, University of Oxford, Oxford, UK; 6 European Synchrotron Radiation Facility, 71 Avenue des Martyrs, 38000 Grenoble, France; 7 The Natural History Museum, Cromwell Road, London, SW7 5BD UK; 8 Department of Organismal Biology, Uppsala University, Norbyvägen 18 A, 752 36 Uppsala, Sweden; 9 Naturalis Biodiversity Center, Darwinweg 2, 2333 CR Leiden, the Netherlands; 10 Forschungs-Neutronenquelle Heinz Maier-Leibnitz, Lichtenbergstr. 1, 85748 Garching, Germany

**Keywords:** Synchrotron tomographic scans, Functional morphology, Jaw mechanism, Ontogeny, Evolution, Triassic

## Abstract

**Background:**

Placodontia is a Triassic sauropterygian reptile group characterized by flat and enlarged crushing teeth adapted to a durophagous diet. The enigmatic placodont *Henodus chelyops* has numerous autapomorphic character states, including extreme tooth count reduction to only a single pair of palatine and dentary crushing teeth. This renders the species unusual among placodonts and challenges identification of its phylogenetic position.

**Results:**

The skulls of two *Henodus chelyops* specimens were visualized with synchrotron tomography to investigate the complete anatomy of their functional and replacement crushing dentition in 3D. All teeth of both specimens were segmented, measured, and statistically compared to reveal that *H. chelyops* teeth are much smaller than the posterior palatine teeth of other cyamodontoid placodonts with the exception of *Parahenodus atancensis* from the Iberian Peninsula. The replacement teeth of this species are quite similar in size and morphology to the functional teeth.

**Conclusion:**

As other placodonts, *Henodus chelyops* exhibits vertical tooth replacement*.* This suggests that vertical tooth replacement arose relatively early in placodont phylogeny. Analysis of dental morphology in *H. chelyops* revealed a concave shape of the occlusal surface and the notable absence of a central cusp. This dental morphology could have reduced dental wear and protected against failure. Hence, the concave teeth of *H. chelyops* appear to be adapted to process small invertebrate items, such as branchiopod crustaceans. Small gastropods were encountered in the matrix close to both studied skulls.

## Background

The extinct Sauropterygia is one of the most diversified clades of Mesozoic marine reptiles that encompasses pachypleurosaurs, nothosaurs, pistosaurs, plesiosaurs, and placodonts [1]. Placodonts are among the first sauropterygians to appear in the fossil record [1, 2]. The first material of Placodontia was described by Münster [3] from the Bavarian Muschelkalk. Since then, its remains were found in Europe [4–9], in the Middle East [10], and in Asia [11–16]. These remains are known from "Oberer Buntsandstein", Anisian [17, 18] to Rhaetian [19] times. Placodonts died out in or just before the Triassic-Jurassic extinction event (~ 201.3 Ma) without any descendants [20]. Their relative rarity and uncertainty regarding the exact age of several placodont-bearing deposits renders reconstruction of the stratigraphical and geographical distribution of Placodontia particularly challenging [21]. Nevertheless, the geological context indicates that placodonts inhabited shallow water near the northeastern and -western Tethys coastal margins [13, 21–23]. This restricted geographical range may reflect their specific diet consisting of hard-shelled prey [24]. Placodonts feature a rather rigid body, a single pair of temporal fenestrae, and a dental architecture (crushing teeth) adapted to a durophagous diet [21, 25].

### Placodontia dentition

Skull reconstructions across placodont diversity permits interspecific comparison [24]. Besides cranial topology, the identification of placodont taxa also strongly relies on dental morphology (e.g., [26–29]). The dentition of placodonts has been studied intensively towards understanding the variability of dental morphology and inferred feeding behavior across this group [2, 24, 27–33]. Tooth location, anatomy, and replacement pattern aids in phylogenetic inferences (e.g., [29, 33]). The dentition and the upper jaw of all major placodont groups are known for their good preservation and can be compared with each other. Teeth are supported by the premaxilla, maxilla, and palatine in the cranium, and the dentary in the mandible. *Paraplacodus* and *Placodus* have long and procumbent teeth, similar to *Palatodonta bleekeri*, on the premaxillary and the anterior part of the dentary [24, 29, 34]. In *Placodus*, these chisel-shaped teeth have a deep root and a more horizontally oriented alveolus [32]. *Cyamodus* and *Protenodontosaurus* are the rare representatives of Cyamodontoidea to bear premaxillary teeth [29, 35]. The maxillary and palatine teeth, in contrast, are adapted for crushing and feature short roots, shallow alveoli, and an ankylosed thecodont tooth implantation [24, 32]. Placochelyid placodonts have fewer teeth than other placodont groups. In *Placochelys* and *Psephoderma*, as in *H. chelyops*, premaxillary teeth (and antagonistic dentary counterparts) are absent in the narrow rostrum [24].

### Placodont phylogeny

Since the end of the twentieth century, the phylogeny of Placodontia has been frequently addressed (e.g., [1, 2, 5, 13–17, 21, 25, 34, 36]). Placodontiformes represents the sister-group to all remaining sauropterygians, the Eosauropterygia [37]. Placodontiformes is the group that includes the non-placodont *Palatodonta bleekeri* as sister taxon to Placodontia [2]. *Paraplacodus* is considered as sister of all remaining placodonts regarding its relatively plesiomorphic dentition and complete lack of osteoderms in the postcranial skeleton [24, 34, 36]. Multiple phylogenetic analyses converged on distinguishing the monophyletic clade Cyamodontoidea from the non-armored placodonts *Paraplacodus* and *Placodus* [1, 5, 16, 34]. Cyamodontoidea is subdivided into the two taxa Cyamodontida and Placochelyida [1, 16]. Cyamodontida is known through *Cyamodus* and *Sinocyamodus* [16]. Placochelyida is composed of the early *Protenodontosaurus italicus* and the two clades Placochelyidae and Henodontidae [16]. The genera *Placochelys*, *Glyphoderma*, *Psephoderma, Psephochelys*, and *Macroplacus* represent Placochelyidae. Henodontidae consists of *Henodus* with the single species *H. chelyops* and the recently described species *Parahenodus atancensis* [8, 16, 38]. *Parahenodus atancensis* is represented only by a single partial cranium. *H. chelyops* shares several characters with other cyamodontoid placodonts, such as a short and broad carapace, cranial tubercles, reducted dentition, and the separation of the nasals with Placochelyidae [24]. *H. chelyops* occurred rather late in stratigraphical deposits, which may imply that this taxon is deeply nested in placodont phylogeny but could also reflect a preservation bias [36].

### *Henodus chelyops* remains

**Fig. 1:**
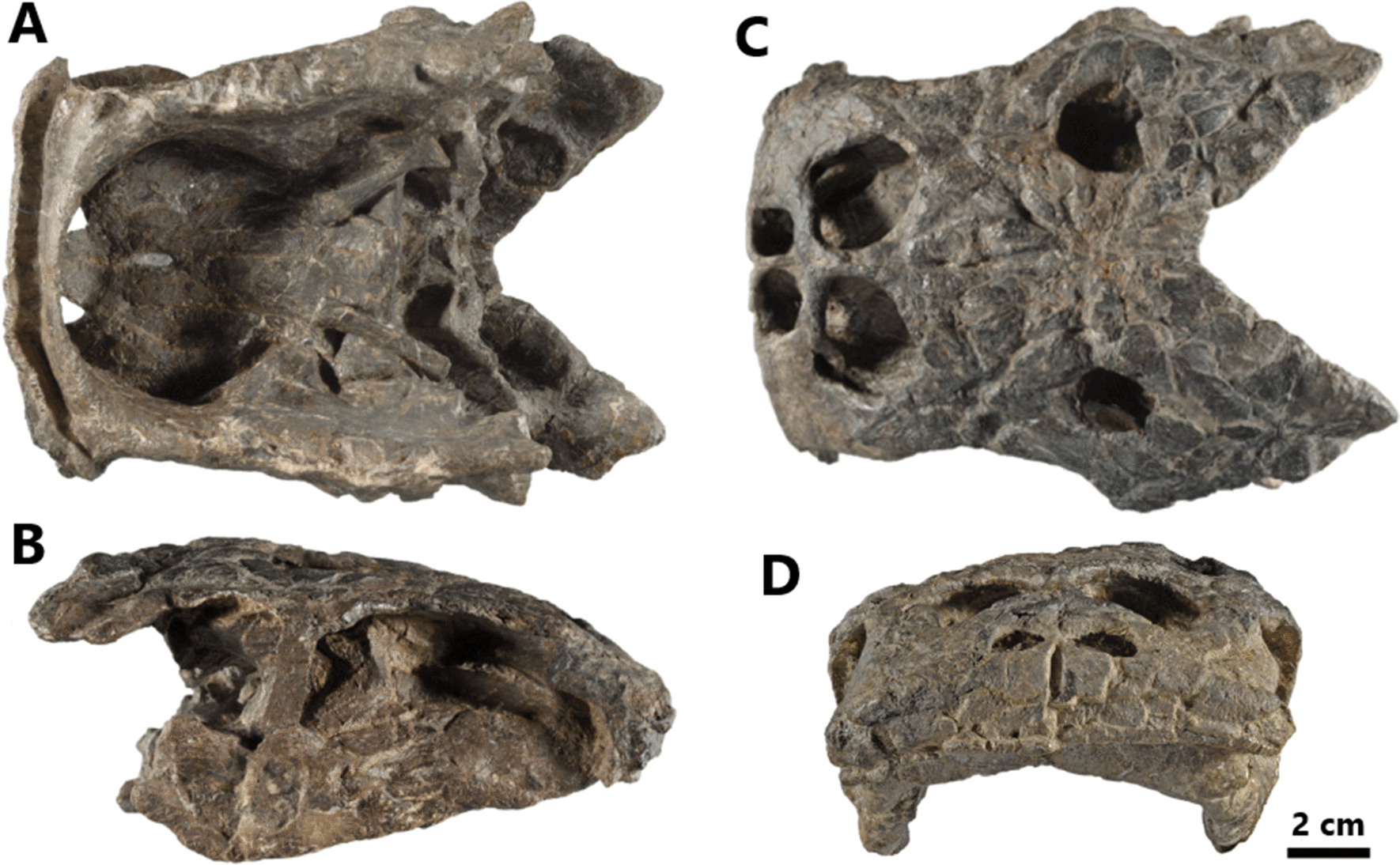
Macroscopic picture of the skull of *Henodus chelyops* specimen GPIT-PV-30003 in ventral (**A**), dextral (**B**), dorsal (**C**) and frontal (**D**) views. The mandible is still articulated with the cranium

*Henodus chelyops* was first assigned to Placodontia by v. Huene in 1936 [18]. All *H. chelyops* remains have been discovered as well-preserved elements at one locality exposing the “Oberer Gipskeuper” of the village of Lustnau near Tübingen (Baden-Württemberg, Germany). These deposits date from the Carnian, and are composed of grey shales with thin silt laminae [18, 39, 40]. These shales have been interpreted as being “ephemeral lacustrine to restricted shallow-marine” deposits [40]. *H. chelyops* represents the only known placodont to have inhabited a brackish lagoonal environment. Several autapomorphic character states were described for this species, such as its roughly rectangular and flat skull, its wide rostrum, and its very deep and massive lower jaw [8, 18, 24, 26, 31, 36, 38, 41, 42] (Fig. 1).

### *Henodus chelyops* dentition

The dentition of *H. chelyops* is extremely reduced [18, 24, 26, 27, 31, 41]. The dentition of *H. chelyops* lacks premaxillary and maxillary teeth and involves a single pair of crushing teeth located across the posterior part of the palatines that occludes with a corresponding pair of teeth across the dentaries. The dental antagonists on the lower jaw are situated medially to the small coronoid processes on the dentary [18]. The pair of teeth on the upper jaw is homologous to the posterior-most palatine teeth of other placodonts [18, 31].

The complex of autapomorphic character states for *H. chelyops* has confounded inference of its phylogenetic position within Placodontia for decades [1, 36]. The atypical ‘dentition’ of *H. chelyops* comprises a unique combination of three structures—a cutting edge with denticles, baleen-like grooves, and crushing palatine teeth—that inspired numerous hypotheses on its feeding behavior that range from filter feeding and suction feeding to herbivory. Durophagy, which is ubiquitous across other placodonts, likely played only a moderate role in this taxon.

### Challenges and aims of the study

The recent development of state-of-the-art tomographic imaging techniques allows for the investigation of internal anatomical features such as the braincase, endocranium, and even replacement teeth [2, 43–45]. Re-examination of well-studied fossils with these non-destructive methods can offer 3D insight in previously inaccessible morphological features. The dentition of *H. chelyops* is rarely visible, as it is typically covered by sediment, or obscured by the cranium and mandibular elements. Recently generated synchrotron tomographic scans of the skulls of several *H. chelyops* individuals are now available in the Paleontological Collection of the University of Tübingen and in the Paleobiology Database of the ESRF. These data expose internal structures of *H. chelyops* crania and mandibles and permit the first extensive study of crushing tooth replacement in *H. chelyops*. The dentition of this taxon has not been examined since Reif and Stein [31].

This study aims to describe the anatomy of the crushing teeth in *H. chelyops* with particular attention to the occlusal surface morphology in *H. chelyops*, and also sets out to explain the tooth replacement pattern of this species relative to those in other placodonts. Based on the above observations, we hypothesize that vertical tooth replacement is a synapomorphy for Placodontia [30, 32, 33, 46]. This dental replacement mode does not seem to have appeared independently among different placodont groups. Tooth replacement has not yet been determined for Henodontidae. Studying the replacement pattern of the reduced palatine crushing teeth in *H. chelyops* will therefore not only provide insight into the ecomorphology and diet of this enigmatic taxon, but will also aid in understanding the evolutionary diversity of dental morphology and replacement strategies across Placodontia.

## Results

### Tooth position and morphology

**Fig. 2:**
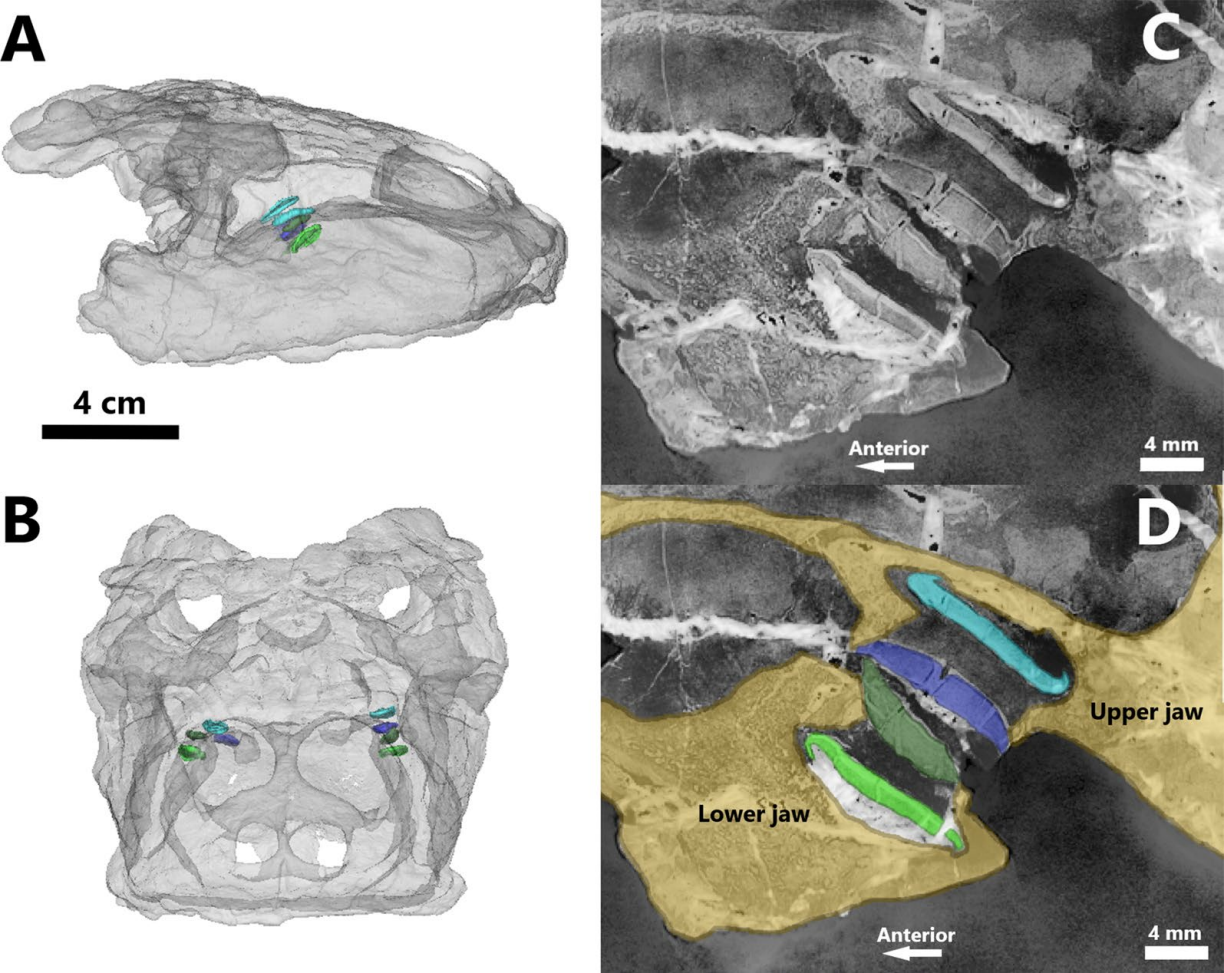
3D model in dextral (**A**) and antero-dorsal (**B**) views of specimen GPIT-PV-30003 exposing the position of the replacement and functional teeth on both upper (teeth in blue) and lower (teeth in green) jaws in *Henodus chelyops*. The replacement teeth are represented with light colors. 75% of transparency was applied to the skull. 2D saggitallice (**C**) and illustration (**D**) allow to obtain more detailed visualization of the position of the teeth

PPC SRµCT data of the skulls of specimens GPIT-PV-30003 and GPIT-PV-30007 uncovered morphological details of *H. chelyops* that are not visible in external view. Thus, it was possible to visualize the internal structure of the skulls but also the functional and replacement crushing teeth. However, cracks, likely filled with minerals rich in metallic elements, are present in several parts of the skulls making it difficult to observe the internal structures. Indeed, they prevent a precise distinction of bone structures. A few teeth are cracked due to poor preservation. All crushing teeth are situated on the posterior part of the palatine and of the dentary. Both specimens carry functional and replacement teeth on the upper and lower jaws (Fig. 2). A single functional crushing tooth is located on each palatine and dentary.

Specimens GPIT-PV-30003 and GPIT-PV-30007 both have a total of four functional teeth, each of which has a corresponding single replacement tooth so that there only is a single generation of tooth replacement in these specimens visible (Fig. 2). Teeth are moderately tilted backward. The tooth replacement is vertical. The replacement tooth is situated in an alveolus deep to the functional tooth. The orientation of the replacement tooth is basically parallel to the functional tooth. The distance between the functional tooth and the associated replacement tooth does not appear to be equivalent between all the teeth of a single specimen. The disparity of the distance between the replacement and the functional tooth observed in this study could illustrate the migration of the replacement tooth and different stages in the replacement process in *H. chelyops*. Otherwise, this disparity could be the result of a taphonomic process. The position of the functional teeth of the upper jaw matches with the functional teeth of the lower jaw (Fig. 2). The functional crushing tooth of the right palatine in specimen GPIT-PV-30003 seems to have moved while burying. It is shifted to the left side and seems not to be attached to the palatine.

**Fig. 3:**
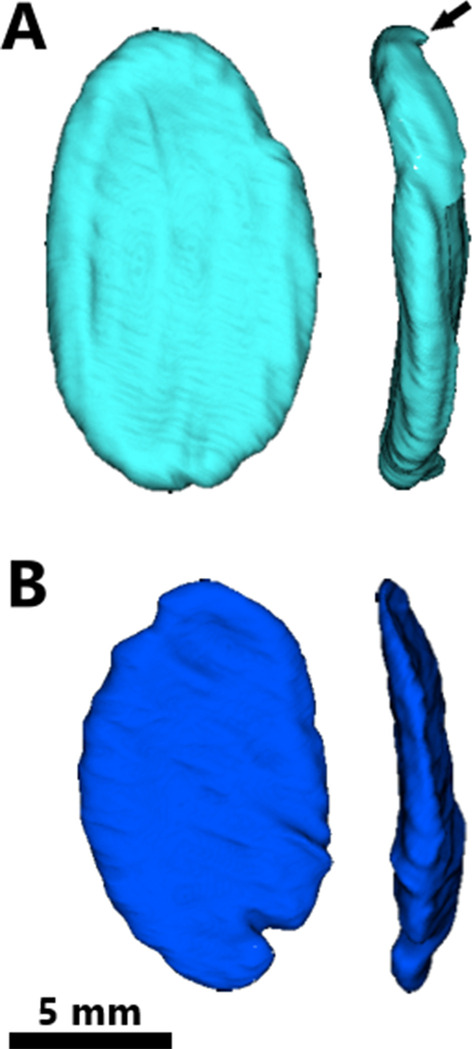
Comparison of the morphology of palatine replacement (A, light blue) and functional (B, blue) teeth in *Henodus chelyops* (specimen GPIT-PV-30007) in occlusal (left) and lingual (right) views. The black arrow shows the short root on the replacement tooth

The crushing teeth in *H. chelyops* are larger in the anteroposterior axis than in the dextrosinistral axis. They generally have an ovoid shape (Fig. 3). The replacement teeth have a similar shape to the functional ones. They are not really flattened as previously mentioned [31] but curved with the occlusal surface being slightly concave. Some slight morphological differences appear between replacement and functional teeth (Fig. 3). Mostly, the thick enamel layer is more marked and larger (around 1 mm) on the replacement teeth than on the functional ones. The progressive wear of the functional teeth can explain why the thin layer of enamel is less observable. The replacement teeth have a little root (Fig. 3) that is less visible for the functional teeth likely due to the ankylosis of the teeth to the alveolar bone.

### Size measurements

The measurements of the length, the width, and the height of each tooth were repeated ten times (see Table 1). The means, the standard deviations, and the standard and relative errors have also been added. For both specimens, the replacement teeth are on average 13.23 ± 1.23 mm long, 8.15 ± 0.59 mm wide, and 2.55 ± 0.19 mm in height (Table 2). The functional teeth are 12.23 ± 1.45 mm long, 7.65 ± 1.05 mm wide, and 2.69 ± 0.17 mm in height (Table 2). In light of these means, the replacement teeth tend to be longer and wider than the functional teeth in these specimens. However, they are smaller in height, which could indicate that the replacement teeth are not fully developed. The relative errors of the tooth length range from 0.052 to 0.405%. The minimum of the relative errors of the tooth width is 0.124% and the maximum is 0.672%. The relative errors of the tooth height are between 0.269% and 1.235%. It should be noted that the measurements of the tooth height are more dispersed than the measurements of the tooth length and width. These differences could be the effect of the positioning of the 3D models. It is necessary to remain prudent with these results. Indeed, the dental wear and the preservation could create a bias on the measured size of the segmented teeth.

**Table 1:** Repeated measurements of the length, the width, and the height of each segmented tooth in specimens GPIT-PV-30003 and GPIT-PV-30007

Measurement	Length 3_1	Width 3_1	Height 3_1	Length 3_2	Width 3_2	Height 3_2	Length 3_3	Width 3_3	Height 3_3	Length 3_4	Width 3_4	Height 3_4
1	12.98	7.64	2.42	10.38	6.7	2.36	9.72	5.68	2.46	11.54	7.3	2.58
2	12.78	7.62	2.32	10.1	6.74	2.28	9.92	5.68	2.36	11.48	7.16	2.46
3	12.94	7.62	2.34	10.46	6.7	2.36	9.82	5.86	2.38	11.46	7.46	2.44
4	12.9	7.66	2.48	10.44	6.82	2.24	9.88	5.72	2.36	11.5	7.46	2.44
5	12.96	7.56	2.34	10.34	6.66	2.36	9.88	5.7	2.42	11.4	7.4	2.44
6	12.96	7.6	2.3	10.2	6.8	2.38	9.82	5.76	2.42	11.3	7.38	2.46
7	12.96	7.62	2.36	10.26	6.64	2.28	9.84	5.68	2.44	11.48	7.62	2.58
8	12.94	7.58	2.28	10.4	6.64	2.34	9.84	5.72	2.42	11.46	7.32	2.52
9	13.02	7.52	2.36	10.44	6.64	2.32	9.8	5.68	2.4	11.14	7.16	2.68
10	12.9	7.54	2.36	10.14	6.66	2.4	10,00	5.52	2.42	11.28	7.14	2.38
Mean (mm)	12.934	7.596	2.356	10.316	6.7	2.332	9.852	5.7	2.408	11.404	7.34	2.498
SD	0.065	0.045	0.058	0.132	0.067	0.051	0.075	0.084	0.033	0.125	0.156	0.091
SE (mm)	0.02	0.014	0.018	0.042	0.021	0.016	0.024	0.027	0.01	0.04	0.049	0.029
RE (%)	0.158	0.187	0.778	0.405	0.315	0.691	0.241	0.468	0.432	0.348	0.672	1.148

Measurement	Length 3_5	Width 3_5	Height 3_5	Length 3_6	Width 3_6	Height 3_6	Length 3_7	Width 3_7	Height 3_7	Length 3_8	Width 3_8	Height 3_8
1	12.94	7.64	2.24	12.26	6.86	2.78	12.4	7.32	2.28	10.58	6.26	2.6
2	13.12	7.9	2.34	12.2	6.86	2.74	12.36	7.18	2.18	10.5	6.34	2.58
3	13.42	7.78	2.34	12.04	6.7	2.64	12.46	7.4	2.22	10.6	6.3	2.5
4	13.42	7.62	2.3	12.16	6.88	2.74	12.24	7.42	2.28	10.46	6.14	2.56
5	13.24	7.84	2.18	12.14	6.64	2.74	12.32	7.24	2.22	10.58	6.28	2.54
6	13.42	7.82	2.24	12.28	6.78	2.52	12.34	7.18	2.38	10.8	6.5	2.58
7	13.4	7.82	2.12	12.12	6.42	2.6	12.2	7.24	2.28	10.8	6.3	2.68
8	13.4	7.62	2.14	12.24	6.72	2.7	12.26	7.46	2.28	10.44	6.2	2.68
9	13.4	7.66	2.12	12.26	6.62	2.6	12.2	7.36	2.32	10.54	6.34	2.64
10	13.4	7.7	2.16	12.18	6.76	2.54	12.36	7.52	2.2	10.52	6.14	2.54
Mean (mm)	13.316	7.74	2.218	12.188	6.724	2.66	12.314	7.332	2.264	10.582	6.28	2.59
SD	0.165	0.104	0.087	0.076	0.14	0.092	0.087	0.119	0.06	0.126	0.107	0.061
SE (mm)	0.052	0.033	0.027	0.024	0.044	0.029	0.027	0.038	0.019	0.04	0.034	0.019
RE (%)	0.393	0.424	1.235	0.196	0.659	1.098	0.223	0.515	0.841	0.376	0.537	0.739

Measurement	Length 7_1	Width 7_1	Height 7_1	Length 7_2	Width 7_2	Height 7_2	Length 7_3	Width 7_3	Height 7_3	Length 7_4	Width 7_4	Height 7_4
1	16,00	9.54	2.92	11.34	8.96	2.6	12.64	8.98	2.72	16.34	9.8	2.58
2	15.98	9.8	2.84	11.56	9.08	2.64	12.5	9.06	2.82	16.3	9.68	2.62
3	15.78	9.8	2.92	11.3	9.02	2.74	12.72	9.06	2.78	16.38	9.66	2.72
4	16.02	9.8	2.88	11.58	9.04	2.72	12.54	8.96	2.78	16.3	9.6	2.44
5	15.88	9.74	2.96	11.58	9.08	2.72	12.64	9.06	2.78	16.32	9.6	2.66
6	15.9	9.74	2.98	11.6	9.1	2.7	12.66	9.06	2.86	16.28	9.68	2.66
7	15.92	9.84	2.92	11.56	9.06	2.7	12.64	9.04	2.84	16.3	9.62	2.68
8	15.92	9.74	2.96	11.58	9.14	2.72	12.64	9.04	2.84	16.3	9.7	2.72
9	15.9	9.74	2.98	11.58	9.08	2.68	12.62	9.02	2.76	16.3	9.6	2.7
10	15.88	9.74	2.9	11.56	9.06	2.7	12.64	9.04	2.82	16.28	9.56	2.68
Mean (mm)	15.918	9.748	2.926	11.524	9.062	2.692	12.624	9.032	2.8	16.31	9.65	2.646
SD	0.07	0.082	0.045	0.109	0.048	0.042	0.062	0.036	0.043	0.03	0.069	0.084
SE (mm)	0.022	0.026	0.014	0.034	0.015	0.013	0.02	0.011	0.014	0.01	0.022	0.027
RE (%)	0.138	0.265	0.489	0.298	0.169	0.498	0.155	0.124	0.488	0.059	0.228	1.008

Measurement	Length 7_5	Width 7_5	Height 7_5	Length 7_6	Width 7_6	Height 7_6	Length 7_7	Width 7_7	Height 7_7	Length 7_8	Width 7_8	Height 7_8
1	14.22	7.8	2.66	15.58	8.02	2.44	11.5	8.7	3.04	13.42	9.26	3.02
2	13.8	7.9	2.96	15.46	7.92	2.52	11.38	8.4	3.18	13.36	9.12	3.06
3	14,00	7.92	2.96	15.44	7.92	2.58	11.5	8.54	3.04	13.38	9.04	3.04
4	14.28	7.9	2.92	15.44	7.94	2.56	11.5	8.56	2.98	13.38	9.06	3.04
5	14.1	7.92	2.92	15.46	7.94	2.56	11.5	8.56	3.02	13.4	9.06	3,00
6	13.94	7.94	2.92	15.44	8,00	2.54	11.52	8.6	3.02	13.38	9.08	3.04
7	14.08	7.94	2.94	15.48	8,00	2.58	11.5	8.66	3,00	13.34	9.1	3.04
8	14.04	7.96	2.94	15.46	7.92	2.58	11.54	8.58	3.04	13.38	9.06	2.98
9	14.08	7.92	2.94	15.44	7.96	2.58	11.52	8.6	3.06	13.38	9.08	3,00
10	14.04	7.96	2.94	15.44	7.92	2.52	11.5	8.66	3.04	13.36	9.04	3,00
Mean (mm)	14.058	7.916	2.91	15.464	7.954	2.546	11.496	8.586	3.042	13.378	9.09	3.022
SD	0.134	0.046	0.089	0.043	0.039	0.044	0.043	0.083	0.054	0.022	0.065	0.026
SE (mm)	0.043	0.015	0.028	0.014	0.012	0.014	0.014	0.026	0.017	0.007	0.02	0.004
RE (%)	0.303	0.184	0.968	0.088	0.155	0.55	0.118	0.307	0.558	0.052	0.225	0.132

The mean, the standard deviation (SD), the standard error (SE), and the relative error (RE) were also added. The measurements were taken on Avizo 8.1. The values are in millimetres.

**Table 2:** Means of the repeated measurements of the length, width, and height of each segmented replacement (repl.) and functional (fct.) tooth in the studied *Henodus chelyops* specimens, as well as the overall mean

Specimen	Length_repl	Width_repl	Height_repl	Length_fct	Width_fct	Height_fct
III	11.4	7.34	2.5	9.85	5.7	2.41
III	12.31	7.33	2.26	10.32	6.7	2.33
III	12.93	7.6	2.36	10.58	6.28	2.59
III	13.32	7.74	2.22	12.19	6.72	2.66
VII	11.5	8.59	3.04	11.52	9.06	2.69
VII	12.62	9.03	2.8	13.38	9.09	3.02
VII	15.46	7.95	2.55	14.06	7.92	2.91
VII	16.31	9.65	2.65	15.92	9.75	2.93
Mean	13.231	8.154	2.547	12.227	7.652	2.692

**Fig. 4:**
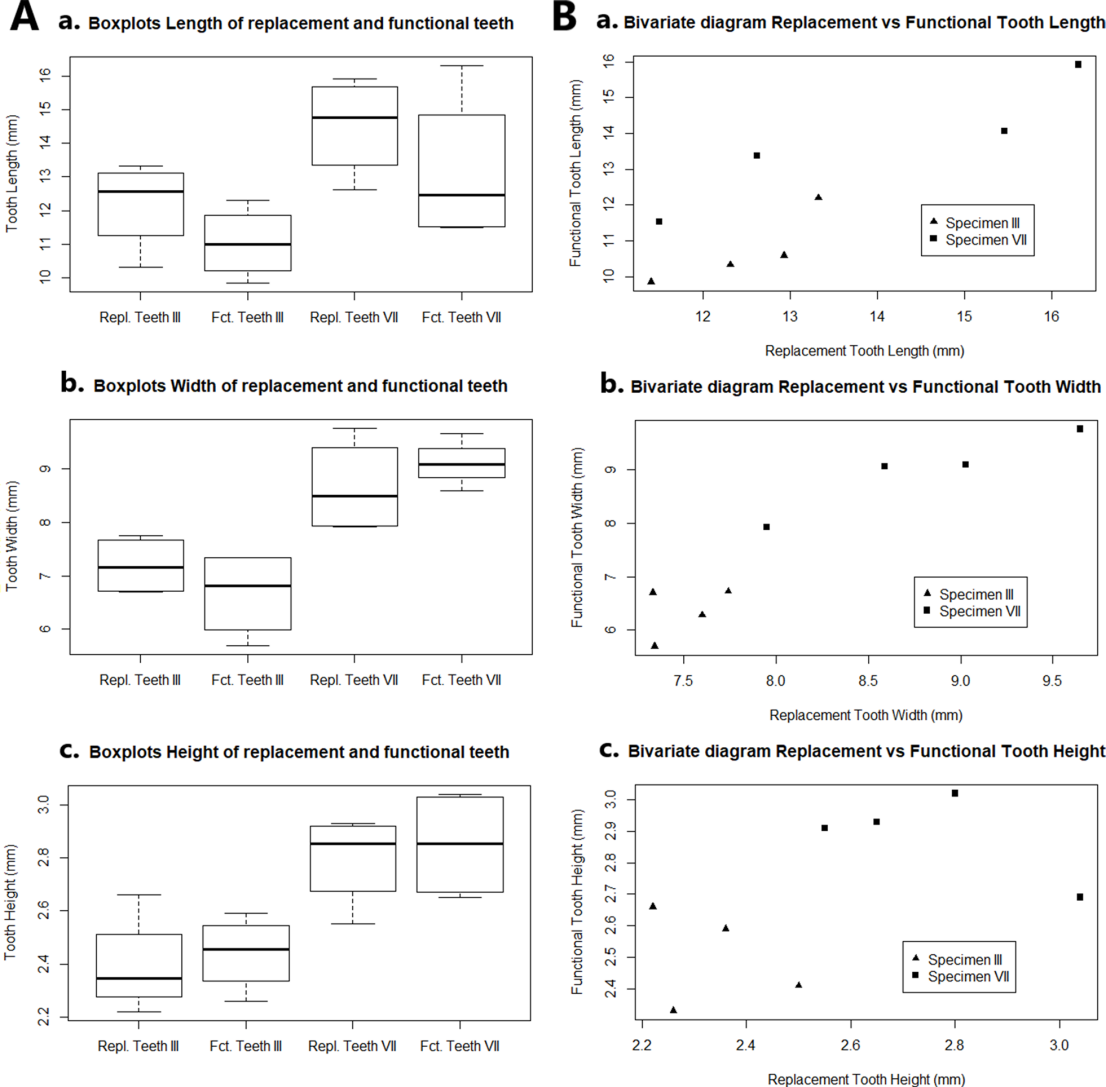
**A** Boxplots comparing the length (**a**), the width (**b**), and the height (**c**) between both specimens GPIT-PV-30003 and GPIT-PV-30007 of *Henodus chelyops* and between their replacement and functional teeth. The thick black lines represent the medians. The values are in millimeters. **B** Bivariate diagrams between the replacement and the functional tooth size in both *H. chelyops* specimens. The length difference (**a**) is illustrated at the top, the width difference (**b**) at the middle and the height difference (**c**) at the bottom. The values of the teeth of specimen GPIT-PV-30003 are represented by triangles and ones of specimen GPIT-PV-30007 are represented by squares. The values are in millimeters

The bivariate diagrams of the length, width, and height of replacement and functional teeth are given in Fig. 4. The values were sorted according to tooth length for each specimen. The size values in specimen GPIT-PV-30007 are generally higher than in specimen GPIT-PV-30003. For example, the tooth width in specimen GPIT-PV-30003 does not exceed 8 mm whereas, in specimen GPIT-PV-30007, it is between approximatively 8 and 10 mm. The smallest replacement and functional teeth in specimen GPIT-PV-30007 have a similar length to those of specimen GPIT-PV-30003. For each specimen, the longer the teeth are, the wider they are, but this is not the case for their height (Fig. 4). The size values for specimen GPIT-PV-30003 are rather clustered when compared to specimen GPIT-PV-30007. The same results were obtained by normalizing the tooth size values with skull length. It was chosen not to mix the values of both specimens for the rest of this study in view of these observations.

The boxplots also reveal the size variation of the functional and the replacement teeth in specimens GPIT-PV-30003 and GPIT-PV-30007, and differences can be observed for the tooth length, width, and height (Fig. 4). The boxplots of the tooth width and height indicate that the size ranges of the replacement teeth of both specimens do not overlap, as shown in the bivariate diagrams. The replacement teeth in specimen GPIT-PV-30007 are wider and higher than in specimen GPIT-PV-30003. It is also the case for the functional teeth. However, the length range of the functional teeth matches with the length range of the replacement teeth in each specimen. The length ranges of the teeth of both specimens overlap. The medians of the tooth width and the height in specimen GPIT-PV-30003 are always lower than in specimen GPIT-PV-30007. Moreover, the median of the length of the replacement teeth is higher than the functional teeth in both specimens.

The resulting p-values from the Mann–Whitney U test to compare the length, the width, and the height between the replacement and functional teeth for each specimen are as follows: in specimen GPIT-PV-30003, the p-value of the tooth length is 0.05714, while the p-value is 0.0294 for the tooth width, and 0.2 for the tooth height. In specimen GPIT-PV-30007, the p-value of the length is 1, 0.6857 for the tooth width, and 0.4678 for the tooth height. The majority of the p-values are greater than 0.05, leading to the acceptance of the null hypothesis that there is no significant size difference between replacement and functional teeth in *H. chelyops*. These high values are partly the result of the low number of tooth samples. However, the widths of the replacement and functional teeth in specimen GPIT-PV-30003 are significantly different with a p-value of 0.0294. It is not the case in specimen GPIT-PV-30007, with a p-value of 0.6857. It is not possible to conclude about a width difference between the functional and the replacement teeth in *H. chelyops*. The p-value of the length difference between the replacement and functional teeth in specimen GPIT-PV-30003 is quite close to 0.05. Even if the p-value is higher than 0.05, this threshold is quite subjective [47]. Thus, the possibility that lengths are significantly equivalent should not be ruled out in particular with regard to the low sample analyzed in this study. There is no confirmation of variation of tooth size either between several specimens in *H. chelyops* nor between functional and replacement teeth in a single individual.

**Fig. 5:**
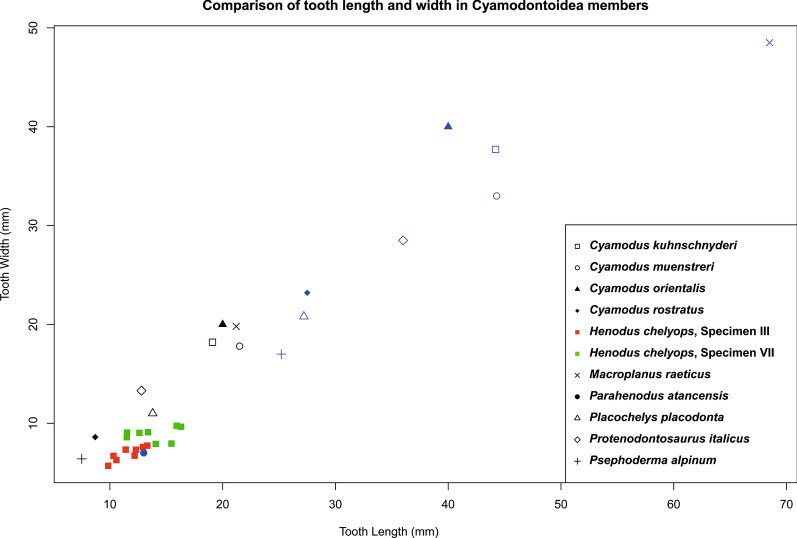
Bivariate diagrams of the tooth length and width in different Cyamodontoidea members. The red and green points, respectively, correspond to the teeth of specimens GPIT-PV-30003 and GPIT-PV-30007 from *Henodus chelyops*. The anterior palatine teeth of other Cyamodontoidea members are represented by black points and the posterior teeth by blue points. The values, in millimeters, are transcribed in Table 3

The measurements of the anterior and posterior teeth of different Cyamodontoidea from the studies of Rieppel and Hagdorn [48], Rieppel [26], Miguel Chaves et al. [8], and Wang et al. [15] were transcribed in Table 3 and plotted in Fig. 5. The mean tooth length and width of each tooth in *H. chelyops* from the present study were also added to this diagram. The length of the posterior palatine teeth in Cyamodontidae and Placochelyidae is always longer than 25 mm and their width wider than 17 mm. In comparison, the palatine teeth in *H. chelyops* have smaller dimensions. The teeth of *H. chelyops* are approximatively half the length and one third of the width of the posterior-most teeth of other Cyamodontoidea. However, the teeth of *H. chelyops* and the anterior palatine teeth of other Cyamodontoidea are similar in length.

**Table 3:** Length and width of the anterior and posterior palatine teeth of different taxa in Cyamodontoidea used in Fig. 5

Taxon	Specimen	Position	Tooth_Length	Tooth_Width
*Cyamodus rostratus*	UMO BT 748 (holotype)	Anterior	8.7	8.6
		Posterior	27.5	23.2
*Cyamodus muensteri*	BMNH R1644	Anterior	21.5	17.8
		Posterior	44.3	33
*Cyamodus kuhnschnyderi*	MHI 1294	Anterior	19.1	18.2
		Posterior	44.2	37.7
*Cyamodus orientalis*	ZMNH M8820 (holotype)	Anterior	20	20
		Posterior	40	40
*Macroplanus raeticus*	BSP 1967 1 324 (holotype)	Anterior	21.2	19.8
		Posterior	68.5	48.5
*Parahenodus atancensis*	MUPA ATZ0104 (holotype)	Posterior	13	7
*Placochelys placodonta*	FAFI Ob/2323/Vt.3 (holotype)	Anterior	13.8	11
		Posterior	27.2	20.8
*Psephoderma alpinum*	MSNM V471	Anterior	7.5	6.4
		Posterior	25.2	17
*Protenodontosaurus italicus*	MFSN 1819GP (holotype)	Anterior	12.8	13.3
		Posterior	36	28.5

Data mainly sourced from Rieppel [26] supplemented with measurements of* Cyamodus kuhnschnyderi* from Rieppel and Hagdorn [48],* Parahenodus atancensis* from Miguel Chaves et al. [8], and* Cyamodus orientalis* from Wang et al. [15]. The values are in millimetres

## Discussion

### Tooth replacement in Placodontia

**Fig. 6:**
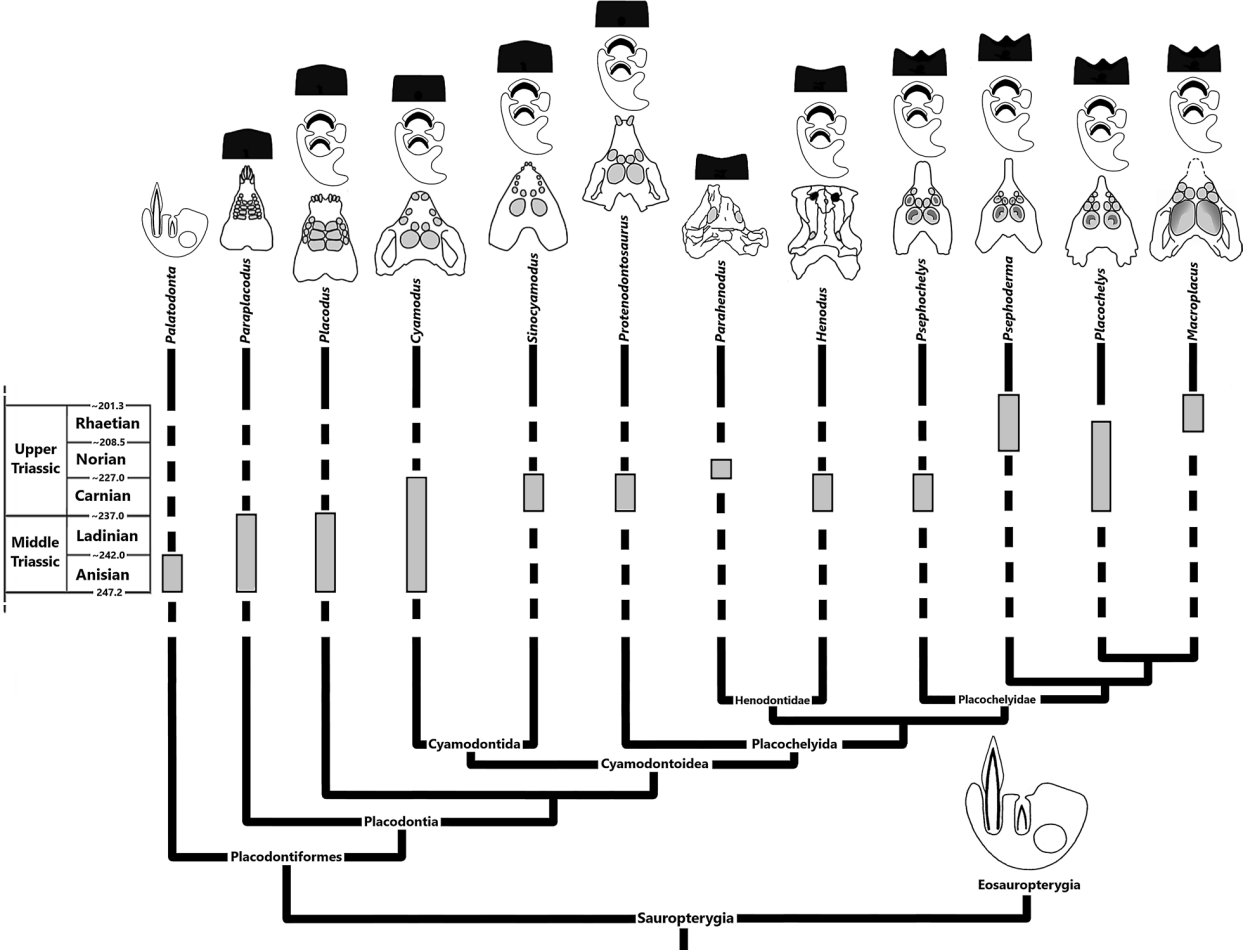
Diversity of the dentition, the tooth replacement, and the palatine tooth morphology inside the placodont phylogenetic tree. The ventral views of the different placodont skulls come from Crofts et al. [29] except for the *Henodus chelyops* skull that was modified from Mazin [24], and the* P. atancensis* skull that was modified from Miguel Chaves et al. [8]. The lingual and vertical tooth replacement representations were made by Rieppel [17]. The lingual tooth replacement is based on his observations on* Nothosaurus* (Eosauropterygia) and the vertical tooth replacement is based on* Placodus gigas* (Placodontia). The occlusal surface shapes were redrawn after Crofts [27, 28] and Crofts et al. [29]. The cladogram is based on the phylogenetic analysis from Wang et al. [16]

Several studies provide discussion on tooth replacement in Sauropterygia [1, 32, 33, 49, 50]. Generally, the replacement tooth grows in distinct alveolar spaces, in a lingual position to the functional tooth in Sauropterygia [32, 33, 50]. Eosauropterygia, such as *Nothosaurus*, has a quite horizontal tooth replacement [32] (Fig. 6), whereas the tooth replacement in *Palatodonta bleekeri* remains unknown [2]. We suggest that this species had a horizontal tooth replacement similar to Eosauropterygia, because it also carries pointed teeth and that vertical tooth replacement is linked to the subsequent flattening of the crushing teeth.

Cranial material of *Paraplacodus* is scarce and poorly preserved to study its tooth attachment and replacement [32, 34]. Following Rieppel [32], in *P. gigas*, the position of the replacement premaxillary teeth is posteroventral for premaxillary teeth and posterodorsal for anterior dentary teeth. This hypothesis is supported by the presence of small opened foramina behind the alveoli of the functional teeth.

As regards the flattened and enlarged crushing teeth in placodonts, the replacement teeth cannot grow in a lingual position to the functional teeth because of their tight spatial arrangement on the maxilla, the palatine, and the posterior part of the dentary [32]. Thin sections exhibit a vertical replacement in *P. gigas*, cyamodontids, and placochelyids [1, 2, 32, 33] (Fig. 6). A large replacement cavity is visible just below the functional tooth, separated by a narrow horizontal shelf (Fig. 2). A wide opening deep to the functional tooth exposes the replacement tooth [32]. When the functional tooth falls out, the horizontal shelf is resorbed by the replacement tooth before it moves upwards into the functional position. This study shows that despite the reduced size of the crushing teeth, vertical tooth replacement is also visible in *H. chelyops* (Fig. 2). Vertical tooth replacement is a character shared by both non-cyamodontoid and cyamodontoid placodonts and could be a potential apomorphy of placodonts within Sauropterygia. In *H. chelyops* and other placodonts, only one generation of tooth replacement was observed. In both studied *H. chelyops* specimens, the replacement teeth seem to have fully developed enamel.

**Fig. 7:**
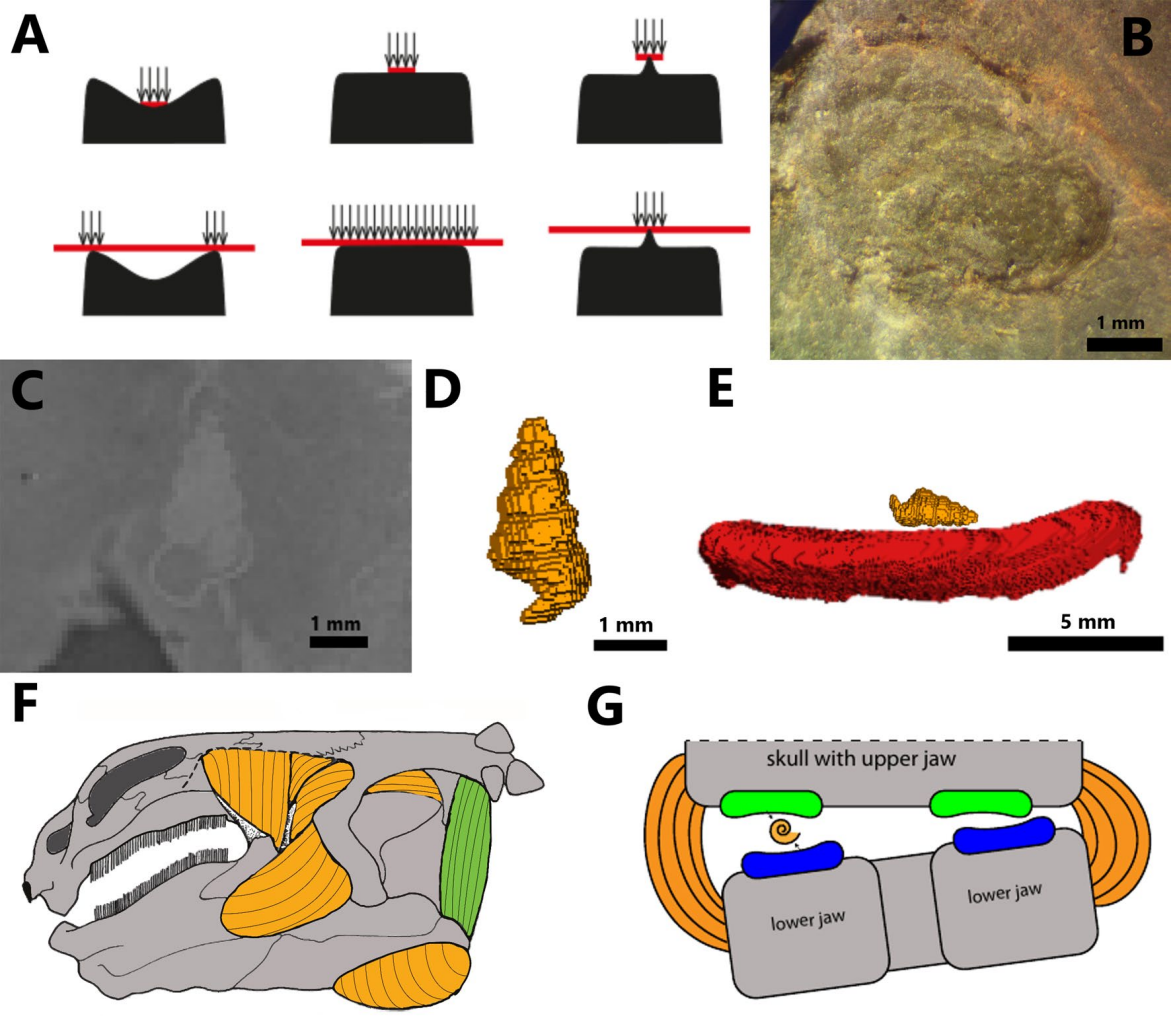
**A** Examples of small and large prey item dental loading regimes according to occlusal surface models: concave (left), flat (middle), and cusped (right), redrawn after Crofts [27, 28]. Red bars and arrows represent the location and direction of applied load for the small (up) and large (down) loading regimes across a representative range of tooth model appearances. **B** Macroscopic picture of the imprint of an *Estheria* shell collected by Reiff [59] (GPIT-PV-30799). *Estheria* is characteristic for the “Gipskeuper Formation” (equivalent to the Grabfeld Formation) in Tübingen/Lustnau where the different *Henodus chelyops* specimens were found. 2D slice (**C**) and 3D representation (**D**) of a gastropod shell found in the matrix attached to the skull of the specimen GPIT-PV-30003 of *H. chelyops*.** E** 3D representation of the gastropod shell on the occlusal surface of the *H. chelyops* tooth. **F** Hypothetical reconstruction of the jaw adductor musculature in *H. chelyops*, redrawn and colorized after Rieppel [3]. The nervus trigeminus innervated muscle is represented in orange and the nervus facialis innervated muscle (m. depressor mandibulae) in green. **G** Reconstruction of the crushing function of the palatine (green) and dentary (blue) teeth in *H. chelyops*. The hypothetical well-developed 1b-portion of musculus adductor mandibulae externus superficialis (orange) could facilitate lateral jaw movement in *H. chelyops*

The unique pattern of tooth replacement in placodonts is the result of phylogenetic trends associated with their durophagous diet [33]. The reduction of the crushing tooth number in advanced placodonts and a particular tooth replacement strategy were selected to make up for a potentially hasty failure of the functional tooth while negotiating spatial constraints [33]. Non-cyamodontoid placodonts experiencing high dental wear exhibit a relatively frequent tooth replacement [27–29, 33]. Indeed, their convex crushing teeth are more prone to dental failure under high loads. No discernable tooth replacement pattern was noticed in early emerging placodonts [33]. The *Placodus* maxillary and palatine teeth are almost simultaneously replaced in order to restrict the breaking down of the separation between juxtaposed replacement alveoli destabilizing the support of the functional teeth [32]. Rieppel [32] considered that the replacement tooth in placodonts is bigger than the preceding functional tooth, especially in juveniles. However, spatial constraints control the maximum size of each tooth generation. In general, it seems as if there were no distinct regularities of tooth replacement rates in Placodontia [32]. Crofts et al. [29] noticed the tooth replacement rates are weaker in placochelyids than in the non-cyamodontoid placodonts despite their low number of maxillary and palatine teeth. In cyamodontoids, the dentition is divided in specific and effective functional units [33]. Teeth are unilaterally replaced in each unit to maintain the crushing function on one side of the mouth. In *H. chelyops*, all palatine and dentary replacement teeth seem to grow at the same time, because a replacement tooth is situated below each functional tooth in both examined *H. chelyops* specimens. It is not possible to conclude about the frequency of the tooth replacement in *H. chelyops* with the little data currently available.

### Placodont tooth morphology

The highly reduced number of crushing teeth in *H. chelyops* sets it apart from other placodonts even if the more advanced placodonts seem to have fewer crushing teeth than the more ancestral ones (Table 4). Our study reports small and oval crushing palatine teeth in *H. chelyops* confirming Reif’s and Stein’s research [31]. The central concavity of the occlusal surface, observed in this study, was not reported by the authors. Nevertheless, they noticed the crushing teeth have a flat crown with a marginal swelling as well as the presence of fine pits. Reif and Stein [31] considered the height of the tooth crown to be 2 mm. For these authors, the palatine teeth in *H. chelyops* are 16–18 mm long and 8 mm wide and the dentary teeth are 13–14 mm long and 7–9 mm wide. These values are approximately the same in our study except for the length of the palatine teeth. The mean length of palatine teeth in specimens GPIT-PV-30003 and GPIT-PV-30007 are 11.61 and 13.85 mm, respectively. Specimen GPIT-PV-30003 has a ratio of tooth length/tooth width of about 1.68 and specimen GPIT-PV-30007 of about 1.57 (Fig. 5). Rieppel [26] also calculated the ratio of the tooth length divided by the tooth width in several species inside the genus *Cyamodus* finding ratios ranging between 1.16 and 1.41, with *Cyamodus tarnowitzensis* having the highest.

**Table 4:** Skull morphological description of different genera included within Placodontia

Taxon		*Paraplacodus*	*Placodus*	*Cyamodus*	*Placochelys*	*Psephoderma*	*Henodus*
Premaxillary teeth	Number & Shape	3 long, procumbent and pointed	3 long, procumbent, spatulated and widely spaced	1 to 2 reduced and blunt	0	0	0
Maxillary teeth	Number & Shape	7 max. low and rounded with a central cusp	3–5 low and rounded (« bulbous») with a central cusp	2–3 hemispherical	3 flattened	2 flattened (posterior one is elliptical)	0
	Size	Quite large	Quite large	Increasing rearwards	Increasing rearwards	Posterior one is larger	-
Palatine teeth	Number & Shape	4 low (3 posterior are rectangular)	3 quadrangular	2–3 circular or elliptical	2 flattened with a central cusp	2 Flattened with a central cusp (posterior one is ovoid)	1 with a flat crown and covered by fine pits
	Size	More large than maxillary teeth	Large	Very big (posterior one is the tallest)	Very big (posterior one is the tallest)	Very big (posterior one is the tallest)	Quite small
Rostrum	Shape	Wide	Wide	Narrow	Narrow, edentulous	Very thin and long, edentulous	Rectangular and wide, denticles

The shape of the rostrum and the number, the shape and the size of teeth (premaxillary, maxillary, and palatine teeth) have been considered. The morphological descriptions come from v. Huene [18], Mazin [24], Reif and Stein [31], and Rieppel [26, 41]

Despite the fragmented skull of the unique specimen (MUPA ATZ0104) attributed to *P. atancensis*, it is possible to analyze its right palatine crushing tooth [8, 38]. The morphology of the palatine teeth in *P. atancensis* and *H. chelyops* is comparable. The right palatine tooth in *P. atancensis* is also ovoid. Miguel Chaves et al. [8] observed a complex dental morphology corresponding to a central concavity with a lateral elevation or crest. The unique entire palatine tooth measures 13 mm in length and 7 mm in width which is quite similar to *H. chelyops* teeth. However, Miguel Chaves et al. [8] considered the palatine teeth in *P. atancensis* to be proportionally bigger than the palatine teeth in *H. chelyops* comparing their length with the width on the articular condyle of the quadrate. Indeed, the length of the palatine teeth in *P. atancensis* is close to the width of the articular condyle of the quadrate, whereas it is less than half in *H. chelyops*.

The palatine teeth have different appearances among placodont groups. In non-cyamodontoid placodonts, they are rather rectangular or quadrangular, whereas in Cyamodontoidea they have a circular, elliptical, or ovoid shape [9, 24, 27–29, 33] (Fig. 6). It is worth noting that Cyamodontoidea literally means “bean-shaped teeth”. The occlusal surface morphology is quite different among the placodonts [27–29] (Fig. 6). Crofts et al. [29] studied the evolution of the occlusal morphology and the “flattening” of the teeth in different placodonts by measuring their radius of curvature.

While the other placodonts have flattened or slightly convex palatine teeth, the occlusal surface of the palatine teeth in Placochelyidae, such as *Placochelys* or *Psephoderma*, and in Henodontoidae is rather concave [29] (Fig. 6). The *Cyamodus* premaxillary and maxillary teeth are more flattened than in *Paraplacodus* and *Placodus*. Even if the caudal-most palatine teeth of some *Cyamodus* specimens (SMNS 91472 and MB.R.1773) are slightly concave, Crofts et al. [29] considered them as functionally flat. Placochelyidae has a distinguished complex palatine tooth morphology with a median cusp and crescent furrows [27–29] (Fig. 6). Finally, it is possible to establish three different tooth morphotypes for the palatine teeth within Placodontia. (A) Non-cyamodontoid and cyamodontid placodonts have palatine teeth with a convex or flattened occlusal surface. (B) Henodontoidae, including *H. chelyops*, have concave palatine teeth without a central cusp. (C) Placochelyidae have concave palatine teeth with a central cusp.

### Feeding strategies and habitat in Placodontia

Generally, it is believed that placodonts fed on bivalves, brachiopods, and maybe decapod crustaceans [20, 37]. These hard-shelled invertebrates are often present in the same deposits as placodonts [20, 27, 51]. This specific diet brought placodonts to stay in shallow water in nearshore environments, which is suggested by the sedimentary facies where they were discovered [20, 24]. Non-cyamodontoid placodonts are only known in epicontinental seas whereas Cyamodontoidea was present in both epicontinental seas and the Tethys [52].

*Henodus chelyops* is known as inhabiting a brackish lagoon environment [21, 25, 41, 52, 53]. The potential food resources are quite scarce in the Oberer Gipskeuper deposits from Tübingen-Lustnau. *Estheria*-crustaceans and some fishes, molluscs, and gastropods are present [31, 39, 52]. The short and oval palatine teeth in *H. chelyops*, its extremely reduced tooth number, and the coronoid process morphology may raise the question of their crushing effectiveness and resistance to hard-shelled molluscs. That is why the majority of the researchers do not consider *H. chelyops* as having had a strongly durophagous diet consisting of thick-shelled molluscan prey items like other placodonts certainly did [8, 20, 27, 31, 37, 41, 42, 54].

The *H. chelyops* skull morphology indicates a low degree of durophagy and bite force. *Henodus chelyops* has been interpreted as herbivorous, as a filter-feeder, or as both [20]. This taxon has been considered herbivorous because of its cutting edge on the premaxilla with the row of tooth-like denticles which could scrape the plants of the substratum [31, 42]. Reif and Stein [31] also considered the grooves with baleen-like structures as a filter or allowing the ejection of superfluous water. Herbivorous marine reptiles are still unusual [42]. Filter-feeding has also been proposed in *H. chelyops* by other authors [8, 18, 27, 41, 55]. The spatulate rostrum would have cut the aquatic vegetation before being filtered out [8]. The rostrum of *H. chelyops* was compared to the spatulate rostrum of *Atopodentatus unicus*, which was interpreted as filter feeder and herbivorous [56, 57]. *H. chelyops* was also declared as a suction feeder due to its massive hyoid apparatus [54]. The extreme tooth reduction of this species is a relevant argument to explain a filtering diet in this species.

The crushing dentition of placodonts represents one of the most extreme forms of durophagy that has ever existed [33]. Durophagy corresponds to the diet of hard-shelled organisms like molluscs or crustaceans. Durophagous animals needed high bite forces [27, 28]. Their feeding habit is associated with different morphological adaptations, such as a change of the teeth position, a robust or heavy skull architecture, or a large temporal skull region for jaw muscle attachments that are found in the more deeply nested placodonts. The crushing teeth of durophagous predators, such as placodonts, have evolved to resist failure against hard-shelled prey [28]. This illustrates how the evolutionary pressure between the prey and the predators can have an influence on the dental morphology. The differences in tooth occlusal morphology indicate that placodonts have evolved different durophagous specializations [29]. It would be worth testing whether the occlusal surface morphology of palatine teeth in *H. chelyops* could prevent a hasty loosening or break of the tooth and indicate whether its dental morphology is adapted to small or large prey items. The likelihood of failure and the pressure applied to break and to crush the prey items are not the same according to the tooth occlusal morphology. The concavity of the occlusal surface, such as in *H. chelyops* palatine teeth, generates a pressure difference when the invertebrate items are crushed [27, 28] (Fig. 7A). If the shell item is small, the pressure is concentrated on the center of the concavity, whereas if the shell item is large, the pressure is put on the marginal border of the occlusal surface (Fig. 7A). For flat teeth, the load of the large prey items is spread over the entire occlusal surface if the prey item is flat [27, 28]. This model hardly ever happens, since the shells of invertebrates are mostly rounded. On the contrary, the loading regime of round hard-shelled items is concentrated in the middle of the occlusal surface of flat teeth. Thus, concave teeth require more force to break shells than flat teeth and convex teeth [27, 28, 58]. However, greater strains were applied to convex teeth that means there is a greater likelihood of crack formation in convex teeth than in concave or flat teeth for large or small prey items [27, 28].

According to the patterns of strain set up by Crofts et al. [27, 28], the concave teeth can undergo ring cracks and edge failures if the strains are too high. This would be a reason why concave tooth appearances are not as common as convex tooth appearances in nature [27, 28]. However, the morphology of concave teeth implies the applied load is distributed over the entire occlusal surface reducing dental wear. Then, the tooth is functional over a longer period, because the enamel stays thick enough to prevent subsurface cracks caused by strains of the invertebrate items. It is also possible to reduce the applied stress on concave teeth if the item has a whorl as seen in gastropods. The concave tooth morphology, such as in *H. chelyops*, increases the area the tooth is in contact with the small items [27, 28]. *H. chelyops* could crush more easily the shelled prey items in the depression of the occlusal surface. The longitudinal crest present in the occlusal surface of the crushing teeth in placochelyids also covers more surface of the shell items than convex or flat teeth and could offset a lack of bite forces in these derived placodonts. In at least some cyamodontids, the repartition of the effective force applied to a shell across the dentition could be balanced.

Multiple small gastropod shells were found within the attached matrix in both *H. chelyops* specimens (Fig. 7C–E). One of these is present close to the right functional dentary tooth and a second specimen was encountered between the right quadrate and the articular of the specimen GPIT-PV-30003. The taxonomic identification of these gastropods was not achieved in this study. These gastropod shells measure around 3 mm in length. This invertebrate looks like a potential candidate as a food source for *H. chelyops* even if these small invertebrates seem to be not very profitable in terms of energy cost and benefit. The small crustacean *Estheria*, which is quite frequent in the deposits from the Grabfeld Formation (formerly “Gipskeuper Formation”) in Germany, could also be preyed upon by *H. chelyops* [59] (Fig. 7B). These hypotheses are admissible only if the deposit environment where the *H. chelyops* remains were found corresponds to its habitat. This has not yet been fully verified [60]. We propose that *H. chelyops* had a multifaceted diet. Thanks to its specific dentition, *H. chelyops* could crush with its palatine teeth invertebrates such as gastropods that are feeding on aquatic vegetation while cropping them with its flat rostrum. These invertebrates could provide supplementary proteins in addition to the aquatic plants and potentially plankton from filter feeding.

The motion of both jaws to crush the invertebrate items with the concave teeth in *H. chelyops* could actually be quite particular. Rieppel [3] studied the jaw musculature of *H. chelyops* based on its wide skull and the specific anatomy of the temporal skull region (Fig. 7F) and concluded that the *H. chelyops* skull does not indicate high bite forces. The hemimandibles are not strongly fused at their symphysis and the articulation between lower jaw and quadrate is relatively complex [26, 34, 61] possibly enabling more intricate movements than just opening and closing of the jaw. A hypothetical 1b-portion of musculus adductor mandibulae externus superficialis, connecting the quadratojugal with the lateral surface of the lower jaw, however, could be well-developed [41] (Fig. 7F and G). With that, an asymmetrical positioning of small prey items in the embayment of the crushing teeth on the left or the right side of the mouth (sensu Druzinsky & Greaves 1979 [62]) and even some lateral movement might have been possible. In this context, the tips of the more fragile edges of the concave teeth [27, 28] would likely not directly contact each other and prevent breakage—similar to the molars of mammals [63] (Fig. 7G).

## Conclusions

New synchrotron CT-scans of the skull of two *H. chelyops* specimens provide novel insights into the diversity of dental morphology and replacement in placodonts. Morphological analysis of the occlusal surfaces of *H. chelyops* teeth revealed that this species carries concave teeth without a central cusp, which are otherwise reported only for *Parahenodus atancensis*. This confirms variable degrees of durophagy within Placodontia. Indeed, its extreme tooth reduction and the small size of its crushing teeth imply that the durophagy is moderate only in henodontid species. The concavity of the occlusal surface in *H. chelyops* appears more adapted to crushing small prey items. This concavity could also be an advantage against the failure likelihood in contrast to convex teeth. It would be important to understand if the different tooth morphotypes within Placodontia are the results of changes in feeding behavior or correspond to different feeding strategies in order to be more efficient to crush hard-shelled prey. The tooth replacement in *H. chelyops* is vertical as observed in *Placodus gigas* and Cyamodontoidea. This study reinforces the hypothesis of generalized vertical tooth replacement across Placodontia. It seems highly improbably that the vertical tooth replacement independently arose in each group. This study has the particular perspective of helping to perceive the most reliable function of the dentition of *H. chelyops* (cutting edges, baleen-like structures, and crushing palatine and dentary teeth).

## Materials & Methods

### Specimens and tomographic scans

v. Huene [18, 64–66], Reiff [53], and Fischer [60] described the eight *H. chelyops* individuals uncovered thus far, which are all stored in the Paleontological Collection of the University of Tübingen. All individuals preserve the skull, except for specimen VIII (GPIT-PV-30008; Roman numerals, as conventional in *Henodus*-literature, designate their original succession of excavation). The well-preserved skulls of specimens III (GPIT-PV-30003) and VII (GPIT-PV-30007) were subjected to synchrotron visualization to be analyzed in this study.

Specimen GPIT-PV-30003, previously freed from the matrix by preparator Wilhelm Wetzel, was described by v. Huene [54]. The carapace and pelvic girdle of this specimen are preserved but the caudal vertebrae are lacking. Although several cracks have propagated the skull of specimen GPIT-PV-30003, it represents the best-preserved *H. chelyops* skull available today. Measuring 16 cm in length and 10.5 cm in width, the skull of specimen GPIT-PV-30003 is slightly compressed on its anterior part and the mandible remained in full articulation with the cranium.

Specimen GPIT-PV-30007, which features a reasonably well-preserved carapace, has not been reported in scientific literature. Its skull has not been prepared before tomographic visualization but its dorsal surface is visible in external view and appears to accommodate upper temporal fenestrae. The skull of specimen GPIT-PV-30007 measures 16 cm in length and maintains the original articulation between cranium and mandible.

Both specimens were characterized using propagation phase contrast synchrotron X-ray micro computed tomography (PPC SRµCT) at the ID17 beamline of the European Synchrotron Radiation Facility (ESRF, Grenoble France). The X-ray setup consisted of: X-ray beam from a wiggler (W150D, gap 26 mm) and a Si 111 double-bent Laue monochromator (set at 120 keV for specimen GPIT-PV-30003 and 132 keV for specimen GPIT-PV-30007). Images were recorded with an indirect detector comprising a 2 mm thick Yttrium Aluminum Garnet (YAG) scintillator, × 0.25 magnification from a set of optical lenses and a FReLoN 2 k charge-coupled device (CCD) camera (using the frame transfer and precision modes). The pixel size for the recorded images of 46.92 µm was obtained measuring the shift of a metallic rod moved using a translation motor perpendicular to the X-ray beam. Given the energy and pixel size, the specimens were placed 11 m in front of the detector to maximise contribution of phase contrast effect. The beam size varied on the vertical axis depending on the energy used: 7.98 × 46.43 mm (vertical x horizontal) for the 120 keV setup and 7.04 × 46.43 mm (v × h) for 132 keV. Slightly different protocols were used for each specimen.

- Specimen GPIT-PV-30007: acquisition of this specimen was more classic (no attenuation or half acquisition protocol), necessitating 43 acquisitions on the vertical axis, moving the sample by 3.8 mm between each acquisition. Each acquisition consisted of 4998 projections of 0.15 s exposure each.
- Specimen GPIT-PV-30003: as the specimen was larger than the field of view, we used the so-called half-acquisition protocol, where the rotation axis of the sample stage is shifted (here shifted by 38.44 mm) to the side of the recorded images [67]. We also used the attenuation protocol, to compensate for the high X-ray attenuation of the specimen [67], plunging the specimen in a 16 cm diameter tube filled with 5 mm diameter aluminum balls. Tomographic acquisitions consisted of 4998 projections of 1 s exposure each, over a 360° of the sample. Thirty-two acquisitions were necessary to compensate for the limited vertical size of the beam, moving the sample by 5.8 mm between each acquisition.

The computed tomography reconstruction was performed with the software PyHST2 [68] using the single distance phase retrieval approach [69]. After the computed tomography reconstruction, post processing of the data included: merging of the dataset along the vertical axis using a weighted average; correction of ring artefact on slices [70]; 32-bit to 16-bit conversion based on 0.002% saturation of the 3D histogram, the data being finally exported as a stack of non-compressed tiff images. To facilitate data handling, a binning 2 × 2 × 2 was generated during the post processing, reducing the data size by 8, generating datasets with an isotropic voxel size of 93.84 µm.

### Morphology and size measurements

We analyzed the functional morphology and replacement mode of the crushing teeth in both *H. chelyops* specimens. The conditions are described and statistically compared to each other and to other placodonts. The stack of tomograms (i.e., slices from the computed tomography reconstruction) was imported in Avizo 8.1 software [71] to segment the teeth and generate 3D rendering. The functional and replacement teeth of both skulls were virtually extracted using manual segmentation in Avizo 8.1 (e.g., Fig. 2). The external surfaces of the skull and of each segmented tooth were converted into a mesh and saved as “PLY” files. These elements were projected as 3D models in the freeware solution MorphoDig 1.5 [72]. Inside their respective skull rendered at 80 percent transparency, each individual tooth was projected opaque to visualize its position and placement.

All replacement and functional teeth of both *H. chelyops* specimens were measured. We assessed whether the replacement teeth were larger than the functional teeth, which is what Rieppel [32] suggested for—especially juvenile—*Placodus gigas* and could imply a gradual increase of placodont tooth size during ontogeny. Anteroposterior length, mediolateral width, and dorsoventral height of each tooth was determined using the 2D Length Tool in Avizo 8.1. Representative locations for the referred dimensions were identified through 3D renderings of the teeth in occlusal (length and width) and lingual view (height; see Fig. 3). Each measurement was conducted ten times to allow for assessment of the measurement error of manual segmentation in both specimens. Furthermore, each set of three complementary measurements was obtained from de novo oriented teeth to mitigate bias in standard orientation. The standard error (SE) was calculated by dividing the standard deviation (σ) over the square root of the number of repeated independent measurements (N): ${\varvec{S}}{\varvec{E}}=\frac{{\varvec{\upsigma}}}{\sqrt{\mathbf{N}}}$. Relative error (RE) was quantified as a percentage of the measured values by dividing the standard error over the mean of the repeated measurements (µ): ${\varvec{R}}{\varvec{E}}=\frac{{\varvec{S}}{\varvec{E}}}{{\varvec{\upmu}}}$. The mean of the repeated measurements of tooth length, width, and height are here considered as the authentic values.

Skull lengths were measured using the 2D Length Tool in Avizo. Dental length, width, and height dimensions of both specimens were plotted in bivariate diagrams capturing the mean values of replacement teeth on the X-axis and the mean values of functional teeth on the Y-axis. These diagrams show whether the different crushing teeth in a single specimen have a homogeneous size. Since the skulls of specimens GPIT-PV-30003 and GPIT-PV-30007 have very similar lengths (158.84 and 158.92 mm, respectively), we could establish whether both *H. chelyops* specimens exhibit dental size differences independent from growth bias. Boxplots showing the distribution of the functional and replacement tooth length, width, and height across the two specimens are also provided. The tooth length, width, and height were also normalized by dividing them by the skull length. Indeed, the older a placodont is, the larger is the size of its crushing teeth might be [32]. Then, the links between length, width, and height of the replacement and functional teeth in *H. chelyops* were tested with the independent 2-group Mann–Whitney U test on R Studio [73, 74]. To have no influence from individuals, this test was carried out independently for both specimens. Finally, the tooth length and width in *H. chelyops* was plotted together with measures of other members of Cyamodontoidea in a bivariate diagram with the length on the X-axis and the width on the Y-axis.

## Data Availability

Scans are available at the server of the Paleontological Collection Tübingen (GPIT) and, upon request, they can be accessed via the curator of the collection. All other data, i.e. measurements, can be found in the Tables.

## Abbreviations

GPIT: former Geologisch-Paläontologisches Institut Tübingen, Germany
PV: Petrefaktenverzeichnis
ESRF: European Synchrotron Radiation Facility
MB.R.: Reptile collection of Museum für Naturkunde, Berlin, Germany
MSNM: Museo Civico di Storia Naturale, Milan, Italy
PPC SRµCT: Propagation phase contrast synchrotron X-ray micro computed tomography
RoC: Radius of curvature
SE: Standard error
RE: Relative error
CCD: Charge-coupled device
YAG: Yttrium aluminum garnet
MUTA APZ: El Atance collection, Museo de Paleontología de Castilla–La Mancha, Spain
